# Estimation of the bidirectional relationship between schizophrenia and inflammatory bowel disease using the mendelian randomization approach

**DOI:** 10.1038/s41537-022-00244-w

**Published:** 2022-03-28

**Authors:** Li Qian, Xiaoyan He, Fengjie Gao, Yajuan Fan, Binbin Zhao, Qingyan Ma, Bin Yan, Wei Wang, Xiancang Ma, Jian Yang

**Affiliations:** 1 Department of Psychiatry, The First Affiliated Hospital of Xi’an Jiaotong University, Xi’an, China; 2 Center for Brain Science, The First Affiliated Hospital of Xi’an Jiaotong University, Xi’an, China; 3 Clinical Research Center, The First Affiliated Hospital of Xi’an Jiaotong University, Xi’an, China

**Keywords:** Schizophrenia, Human behaviour

## Abstract

It has been reported that schizophrenia (SCZ) and inflammatory bowel disease (IBD) are related. However, whether there is a bidirectional interaction between them remains unclear. The aim of this study was to conduct a bidirectional Mendelian randomization (MR) analysis to elucidate the causal relationship between SCZ and IBD and its subtypes, including Crohn’s disease (CD) and ulcerative colitis (UC). Single-nucleotide polymorphisms (SNPs) extracted from the summary data of genome-wide association studies were used as genetic instruments. MR was performed using the inverse-variance-weighted method. The MR-Egger and weighted median methods were used for sensitivity analyses. Analysis using 70 SNPs as genetic instruments showed that SCZ was associated with an increased risk of IBD (OR = 1.14, 95% CI: 1.09–1.20, *P* = 9.21 × 10^−8^), CD (OR = 1.16, 95% CI: 1.07–1.25, *P* = 1.42 × 10^−4^), and UC (OR = 1.14, 95% CI: 1.07–1.21, *P* = 2.72 × 10^−5^). The results of the sensitivity analyses were robust and no evidence of pleiotropy was observed. Bidirectional MR analyses showed no causal effects of IBD, CD, or UC on SCZ. This study suggests that SCZ has causal effects on IBD and its subtypes, whereas IBD has no effect on SCZ. Brain-gut axis interactions may help clarify the causal relationship between SCZ and IBD. However, further studies are needed to elucidate the biological mechanisms behind the brain-gut interactions.

## Introduction

Schizophrenia (SCZ) and inflammatory bowel disease (IBD) are responsible for a substantial proportion of cases of disability in the general population worldwide^1^. SCZ is a chronic psychiatric disorder that mainly manifests as cognitive and behavioral abnormalities^2^, whereas IBD is characterized by immune dysregulation and inflammation of the gut^3^. Observational epidemiological investigations have indicated that patients with SCZ have an increased risk of developing IBD^4^ and vice versa^5,6^. Emerging genetic evidence also shows that there is a genetic association between the two diseases^7,8^. However, whether there is a bidirectional interaction between SCZ and IBD remains unclear.

Mendelian randomization (MR) is an alternative tool for identifying causal associations between modifiable exposure and disease outcomes using genetic variants as instrumental variables^9^. The fundamental framework of the MR study design is that if genetic variants can predict the level or biological effect of a modifiable exposure to some extent, then they should also be causally associated with the exposure-related disease outcome to the same extent that they act on the exposure^10^. Utilizing the fact that genetic variants are randomly assigned before birth and fixed at conception, the MR design can prevent confounding, reverse causation, and various biases that are common in observational investigations^11^. In addition, the considerable increase in the number of publicly available genome-wide association studies (GWASs) has provided abundant data sources and increased the statistical power of MR. This makes the MR very popular for elucidating causality.

In this study, we conducted a bidirectional MR analysis to elucidate the causal relationship between SCZ and IBD and its subtypes, including ulcerative colitis (UC) and Crohn’s disease (CD). We hypothesized that there is a bidirectional causal interaction between SCZ and IBD.

## Results

**Fig. 1:**
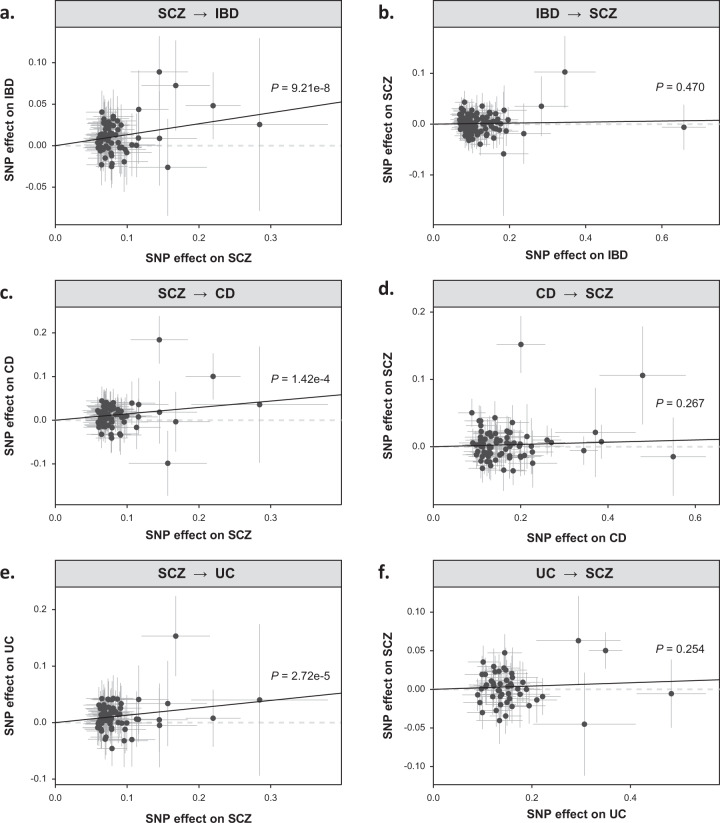
Scatterplot of genetic associations between SCZ and IBD and its subtypes. Scatter plots show the MR-derived associations between genetically predicted (**a**) SCZ on IBD; **b** IBD on SCZ; **c** SCZ on CD; **d** CD on SCZ; **e** SCZ on UC; **f** UC on SCZ. SCZ: schizophrenia. Associations are calculated using the inverse-variance weighted method. SCZ Schizophrenia, IBD Inflammatory bowel disease, CD Crohn’s disease, UC Ulcerative colitis.

Using 70 single-nucleotide polymorphisms (SNPs) (*R*^2^ = 3.5%; *F* = 42.8; Supplementary Table 1) as genetic instruments, the inverse-variance weighted (IVW) MR analysis showed that SCZ was associated with an approximately 14% increased risk of developing IBD (odds ratio [OR] = 1.14, 95% confidence interval [CI]: 1.09–1.20, *P* = 9.21 × 10^−8^; Table 1; Fig. 1a). The causal inference of the sensitivity analysis conducted using the MR-Egger method (OR = 1.25, 95% CI: 1.03–1.50, *P* = 0.025), the weighted median method (OR = 1.13, 95% CI: 1.06–1.20, *P* = 1.25 × 10^−4^) and the MR-PRESSO method (OR = 1.14, 95% CI: 1.09–1.20, *P* = 1.12 × 10^−6^) was robust. The results of the MR-Egger intercept test suggested no evidence of horizontal pleiotropy (*P* = 0.350). Significant causal effects of SCZ on CD (IVW OR = 1.16, 95% CI: 1.07–1.25, *P* = 1.42 × 10^−4^; Table 1; Fig. 1c) and UC (IVW OR = 1.14, 95% CI: 1.07–1.21, *P* = 2.72 × 10^−5^; Table 1; Fig. 1e) were also observed. The MR-Egger intercept test showed no evidence of horizontal pleiotropy.

**Table 1:** Mendelian randomization estimates for causal effects of genetically predicted SCZ on IBD and its subtypes.

Method	No. of SNPs^a^	MR analysis			MR-Egger Intercept *P*
		OR	95% CI	*P*	
*SCZ* → *IBD*					
IVW	70	1.14	1.09 to 1.20	9.21e-08	0.350
MR-Egger	70	1.25	1.03 to 1.50	0.025	
Weighted Median	70	1.13	1.06 to 1.20	1.25e-04	
MR-PRESSO^b^	70	1.14	1.09 to 1.20	1.12e-06	
*SCZ* → *CD*					
IVW	70	1.16	1.07 to 1.25	1.42e-04	0.171
MR-Egger	70	1.41	1.06 to 1.88	0.023	
Weighted Median	70	1.15	1.06 to 1.25	7.64e-04	
MR-PRESSO^b^	70	1.13	1.06 to 1.21	5.22e-04	
*SCZ* → *UC*					
IVW	70	1.14	1.07 to 1.21	2.72e-05	0.996
MR-Egger	70	1.14	0.90 to 1.45	0.284	
Weighted Median	70	1.10	1.01 to 1.19	0.021	
MR-PRESSO^b^	70	1.14	1.07 to 1.21	7.97e-05	

*Note:* *MR* Mendelian randomization, *SCZ* Schizophrenia, *IBD* Inflammatory bowel disease, *CD* Crohn’s disease, *UC* Ulcerative colitis, *SNPs* Single-nucleotide polymorphisms.

^a^SNPs are selected at the genome-wide significant threshold of *P* < 5 × 10^−8^ with a linkage disequilibrium threshold of r^2^ < 0.001 in a 10-Mb window.

^b^No outliers have been detected for MR estimates of SCZ on IBD, CD, and UC.

Table 2 shows the results of the causal effects of IBD, CD, and UC on SCZ. Ninety-eight (*R*^2^ = 11.2%; *F* = 70.0; Supplementary Table 2), 75 (*R*^2^ = 15.0%; *F* = 94.3), and 50 (*R*^2^ = 7.7%; *F* = 76.4) SNPs were extracted for IBD, CD, and UC, respectively. However, based on these SNPs, no causal effects of IBD (IVW OR = 1.01, 95% CI: 0.98–1.04, *P* = 0.470; Fig. 1b), CD (IVW OR = 1.02, 95% CI: 0.99–1.05, *P* = 0.267; Fig. 1d) and UC (IVW OR = 1.02, 95% CI: 0.98–1.06, *P* = 0.254; Fig. 1f) on SCZ were observed. Sensitivity analyses also showed consistent results, suggesting that IBD and its subtypes have no causal effects on SCZ.

**Table 2:** Reverse Mendelian randomization estimates for causal effects of genetically predicted IBD and its subtypes on SCZ.

Method	No. of SNPs	MR analysis			MR-Egger Intercept *P*
		OR	95% CI	*P*	
*IBD* → *SCZ*					
IVW	98	1.01	0.98 to 1.04	0.470	0.464
MR-Egger	98	0.99	0.92 to 1.06	0.690	
Weighted Median	98	1.01	0.97 to 1.04	0.727	
MR-PRESSO^b^	97	1.01	0.99 to 1.04	0.286	
*CD* → *SCZ*					
IVW	75	1.02	0.99 to 1.05	0.267	0.592
MR-Egger	75	0.99	0.92 to 1.08	0.923	
Weighted Median	75	1.02	0.99 to 1.05	0.218	
MR-PRESSO^b^	72	1.01	0.98 to 1.03	0.629	
*UC* → *SCZ*					
IVW	50	1.02	0.98 to 1.06	0.254	0.586
MR-Egger	50	1.05	0.94 to 1.17	0.375	
Weighted Median	50	1.01	0.97 to 1.04	0.800	
MR-PRESSO^b^	45	1.01	0.97 to 1.04	0.788	

*Note:* *MR* Mendelian randomization, *SCZ* Schizophrenia, *IBD* Inflammatory bowel disease, *CD* Crohn’s disease, *UC* Ulcerative colitis, *SNPs* Single-nucleotide polymorphisms.

^a^SNPs are selected at the genome-wide significant threshold of *P* < 5 × 10^−8^ with a linkage disequilibrium threshold of r^2^ < .001 in a 10-Mb window.

^b^One outlier has been detected for MR estimate of IBD on SCZ, three outliers for CD on SCZ, five outliers for UC on SCZ.

## Discussion

In this study, we conducted a bidirectional MR analysis to assess the causal relationship between SCZ and IBD using GWAS summary-level data. Our findings provide genetic evidence that SCZ has causal effects on IBD and its subtypes, whereas IBD has no causal effect on SCZ. To the best of our knowledge, this is the first MR study to elucidate the causal relationship between SCZ and IBD.

The psychiatric comorbidities of IBD are well known. However, whether psychiatric factors cause IBD or whether IBD has an impact on psychiatric disorders has not yet been determined. The present study provides evidence that SCZ has causal effects on IBD and its subtypes, which supports the idea that psychiatric factors play a role in the development of IBD. Indeed, psychiatric symptoms, such as anxiety, have been reported to be associated with an increased risk of surgery and disease relapse, poorer quality of life, and increased likelihood of using immunomodulators in patients with IBD^12–15^. In addition, a nationwide study indicated that patients diagnosed with SCZ have an elevated incidence rate of IBD compared with those without SCZ^4^. Moreover, a growing body of evidence suggests that patients with IBD could benefit from psychological treatments^16–18^. Furthermore, some clinical experts have recommended including psychiatric symptom scales in the routine screening of IBD. These previous reports highlight the significance of psychiatric factors in the diagnosis and treatment of IBD^19^.

The findings of the present study do not support the idea that IBD has a causal effect on SCZ. This is inconsistent with the findings of several previous studies^5,6^. There may be several reasons for this discrepancy. First, although psychiatric comorbidities are common in IBD, psychiatric disorders in most cases of IBD are underdiagnosed. In addition, some psychiatric problems may have occurred years prior to the diagnosis of IBD^20^. Second, observational studies are easily biased by confounding factors. Some unknown factors, such as psychotropic substance abuse, infection, or psychological trauma, may be responsible for the elevated incidence of SCZ among patients with IBD^21,22^. Third, the present study demonstrated that genetic determinants of SCZ have a significant impact on IBD. However, some genetic mediators may lead to the development of IBD before the diagnosis of SCZ. A recent general population-based study showed no evidence of a correlation between IBD and an increased risk of SCZ, a result that supports the findings of our study^23^. Interestingly, a recent bidirectional MR study of depression and IBD also suggested that depression was associated with a higher risk of IBD. While in contrast, no causal effect was observed of IBD and depression, which was consistent with the findings of our study^24^.

The brain-gut axis is believed to play an essential role in elucidating the underlying biological mechanisms linking SCZ and IBD^25–27^. Psychological representations may influence gastrointestinal function through the generation of a stress response, activation of the neuroendocrine system, or stimulation of the autonomic nervous system^28^. The gut microbiome is also involved in a number of brain processes, such as stress hormone signaling, neural function, and neuroprotection^29^. Bidirectional brain-gut axis interaction is a plausible explanation for the occurrence of psychiatric comorbidities in cases of IBD^26^. However, the results of the present study do not support the idea of a bidirectional interaction between SCZ and IBD, suggesting that bidirectional communication along the brain-gut axis is not specifically reversible. The impact of SCZ on gastrointestinal function is more direct, whereas IBD cannot lead to SCZ through a specific brain-gut pathway.

This study has several strengths. First, the effects of unmeasured confounders and reverse causation were avoided through the use of MR design and data from large GWASs. Second, the large sample sizes of the GWASs utilized for this study strengthened the power of the causal inferences from the summary data-based MR analysis. Third, the sensitivity analyses and pleiotropy tests conducted using multiple MR methods provided a robustness evaluation of the MR estimates.

There were also limitations to this study. First, most of the participants in the included study samples were of European ancestry. This might limit the generalizability of the study findings to other populations. In fact, the genetic architecture of SCZ in East Asian populations is very different from that in European populations^30^. In contrast, the incidence and prevalence of IBD among Asians are significantly lower than those among Europeans^31^. Therefore, the causal relationship between SCZ and IBD needs to be studied in other populations. Second, although MR is an effective tool for elucidating causality without the interference of environmental confounders, it is known to be susceptible to horizontal pleiotropy. We adopted several methods of sensitivity analysis to control for horizontal pleiotropy. However, we could not completely exclude bias from pleiotropy, which could reduce the validity of the results. Third, although brain-gut interactions may help reveal the biological mechanisms underlying the relationship between SCZ and IBD, further studies are needed to determine the specific process or pathway of gut-brain axis interactions.

In conclusion, this study provides genetic evidence that SCZ has causal effects on IBD and its subtypes, whereas IBD has no effect on SCZ. Brain-gut axis interactions may aid the understanding of such associations. However, the specific biological mechanisms behind the interactions need to be explored further.

## Methods

### Study design

**Fig. 2:**
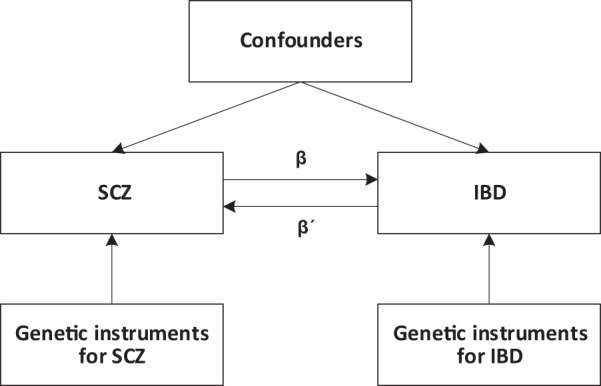
Bidirectional Mendelian randomization study design. β is the causal effect of genetic instruments of SCZ on IBD, whereas β´ is the causal effect of genetic instruments of IBD on SCZ. SCZ schizophrenia, IBD inflammatory bowel disease.

We employed a bidirectional MR study design to estimate the causal relationship between SCZ and IBD (Fig. 2. MR analysis was first performed in one direction to determine the causal effect of SCZ on IBD. Thereafter, the analysis was performed in the opposite direction. All analyses were performed using summary-level data from publicly available GWASs. Therefore, no ethical approval and consent were required for this study.

### Data source and instruments

#### Data for schizophrenia

Information on the genetic associations with SCZ were obtained from the GWAS by the Psychiatric Genomics Consortium (PGC)^32^. The PGC conducted the most comprehensive GWAS on SCZ, including 36,989 cases and 113,075 controls selected from 46 European and three East Asian cohorts. Cases were diagnosed according to the criteria in the Diagnostic and Statistical Manual of Mental Disorders (DSM)-III or DSM-IV, after an interview by a psychiatrist and review of medical records. Genotypes were gathered from each cohort and were processed by the PGC using unified quality control procedures. Association meta-analysis was conducted using an inverse-weighted fixed-effects model, after adjusting for the first ten principal components.

#### Data for inflammatory bowel disease

Summary data for IBD, UC, and CD were derived from the study by the International Inflammatory Bowel Disease Genetics Consortium^33^. The study participants comprised 25,042 cases and 34,915 controls for IBD, 12,366 cases and 33,609 controls for UC, and 12,194 cases and 28,072 controls for CD. All the study participants were of European ancestry, and all cases were diagnosed using accepted endoscopic, histopathological, and radiological criteria. Association tests were performed using an additive frequentist model conditioned on the first ten principal components for each cohort, followed by a meta-analysis using the weighted standard error method.

#### Instrument selection criteria

Genetic instruments for SCZ and IBD were extracted using the same criteria. We selected all relevant SNPs at the genome-wide significance (*P* < 5 × 10^−8^) threshold from each GWAS, and pruned for independence using a clumping procedure in PLINK v1.9 (http://www.cog-genomics.org/plink/1.9/), setting a linkage disequilibrium threshold of r^2^ < 0.001 in a 10-Mb window. Datasets for exposure and outcome were then harmonized, and palindromic SNPs with intermediate allele frequencies were excluded. For SNPs absent in the outcome dataset, a proxy SNP (r^2^ > 0.8) was used or discarded if no proxy was available. Two parameters, the proportion of variance explained by the SNPs (*R*^2^) and *F* statistics, were used to evaluate the strength of the selected instrument^34^. Typically, an *F* statistic >10 is considered sufficiently informative for MR analyses^35^.

### Power calculations

Power calculations were performed using the online tool mRnd (http://cnsgenomics.com/shiny/mRnd/). There were 87% power to detect a relative 10% difference (an OR of at least 1.10 or 0.90) in risk of SCZ on IBD, and 97% power to detect a 10% difference in risk of IBD on SCZ.

### Statistical analysis

The MR analysis was conducted in two directions. The first was the causal effect of genetically predicted SCZ on IBD, whereas the second was the reverse effect of genetically predicted IBD on SCZ. The IVW method was used for the standard MR analysis, in which all genetic variants were assumed to be valid instruments. Therefore, the MR provided a true slope of SNP-outcome association on SNP-exposure association with the intercept constrained to zero^36^. However, the IVW method is vulnerable to horizontal pleiotropy^37^. Thus, for the sensitivity analysis, we utilized several additional methods, such as the weighted median, the MR-Egger, and the MR-PRESSO methods, which are insusceptible to horizontal pleiotropy. The weighted median method can provide a consistent estimate when up to 50% of the SNPs are invalid instruments^38^, whereas the MR-Egger method works even when all SNPs are invalid^39^. The MR-PRESSO is a newly developed method that has the ability to detect and correct for horizontal pleiotropic outliers^40^. Together, these methods provide a robustness test of the causal estimate derived from the MR analysis^41^. Besides, the MR-Egger intercept test was used to detect whether horizontal pleiotropy existed^39^. All analyses were performed using the “TwoSampleMR” and the “MR-PRESSO” packages in R v3.61 (https://www.r-project.org/). Statistical significance was set at *P* < 0.05.

## Supplementary information

Supplemental Material

## Data Availability

Publicly available datasets are analyzed in this study. GWAS summary data for SCZ are publicly available at the PGC website (https://www.med.unc.edu/pgc). GWAS datasets of IBD, CD, and UC can be downloaded from the GWAS Catalog (https://www.ebi.ac.uk/gwas/).
