# Supplementary material for: Estimation of the bidirectional relationship between schizophrenia and inflammatory bowel disease using the mendelian randomization approach
Source: Schizophrenia (Heidelb). 2022 Mar 28;8(1):31. doi: 10.1038/s41537-022-00244-w (PMC9261100; doi:10.1038/s41537-022-00244-w)
Supplement: Supplementary file 1 — Supplemental Material [file 41537_2022_244_MOESM1_ESM.docx]

**Estimation of the** **Bidirectional Relationship Between Schizophrenia and** **Inflammatory Bowel Disease Using the Mendelian Randomization Approach**

Li Qian, Xiaoyan He, Fengjie Gao, Yajuan Fan, Binbin Zhao, Qingyan Ma, Bin Yan, Wei Wang, Xiancang Ma, Jian Yang

**Supplemental Table 1. Genetic instruments for SCZ and associations with IBD, CD and UC.**

| **SNP** | **Proxy SNP** | **r2 for proxy** | **R^2^** | **A1 (A2)** | **β (SE) for SCZ** | **β (SE) for IBD** | **β (SE) for CD** | **β (SE) for UC** |
| --- | --- | --- | --- | --- | --- | --- | --- | --- |
| rs10520163 | - | - | 4.01E-04 | C(T) | -0.06(0.01) | -0.01(0.01) | 0.01(0.02) | -0.01(0.02) |
| rs10791097 | - | - | 5.90E-04 | G(T) | -0.07(0.01) | -0.01(0.01) | -0.01(0.02) | -0.01(0.02) |
| rs11027857 | - | - | 4.24E-04 | A(G) | 0.06(0.01) | 0.01(0.01) | 0.01(0.02) | 0.01(0.02) |
| rs1106568 | - | - | 3.93E-04 | A(G) | -0.07(0.01) | 0.02(0.01) | 0.02(0.02) | 0.03(0.02) |
| rs11210892 | - | - | 4.68E-04 | A(G) | -0.07(0.01) | -0.01(0.01) | -0.01(0.02) | -0.01(0.02) |
| rs11682175 | - | - | 5.95E-04 | C(T) | 0.07(0.01) | 0.02(0.01) | 0.01(0.02) | 0.03(0.02) |
| rs11693094 | - | - | 5.70E-04 | T(C) | -0.07(0.01) | -0.01(0.01) | -0.01(0.02) | -0.01(0.02) |
| rs117074560 | - | - | 3.88E-04 | T(C) | -0.16(0.03) | 0.03(0.03) | 0.1(0.04) | -0.03(0.04) |
| rs12129573 | - | - | 4.83E-04 | A(C) | 0.07(0.01) | 0.01(0.01) | 0.02(0.02) | 0.01(0.02) |
| rs12691307 | - | - | 5.00E-04 | G(A) | -0.07(0.01) | 0.01(0.01) | -0.03(0.02) | 0.03(0.02) |
| rs12704290 | - | - | 5.07E-04 | A(G) | -0.11(0.02) | -0.01(0.02) | -0.04(0.03) | 0.03(0.02) |
| rs12826178 | - | - | 5.78E-04 | T(G) | -0.17(0.02) | -0.07(0.03) | 0.01(0.03) | 0.01(0.04) |
| rs12887734 | - | - | 6.73E-04 | T(G) | 0.09(0.01) | 0.02(0.01) | 0.02(0.02) | 0.01(0.02) |
| rs12903146 | - | - | 4.54E-04 | A(G) | 0.06(0.01) | 0.01(0.01) | 0.02(0.02) | 0.01(0.02) |
| rs13217619 | - | - | 1.54E-03 | C(T) | -0.22(0.02) | -0.05(0.02) | -0.1(0.03) | -0.01(0.03) |
| rs13240464 | - | - | 6.29E-04 | C(T) | -0.08(0.01) | -0.03(0.01) | -0.03(0.02) | -0.03(0.02) |
| rs140505938 | - | - | 4.57E-04 | T(C) | -0.09(0.01) | 0.01(0.02) | 0.03(0.02) | -0.03(0.02) |
| rs1498232 | - | - | 4.49E-04 | C(T) | -0.07(0.01) | -0.02(0.01) | -0.03(0.02) | -0.01(0.02) |
| rs1501357 | - | - | 3.96E-04 | C(T) | 0.08(0.01) | 0.02(0.02) | 0.02(0.02) | 0.01(0.02) |
| rs16867576 | - | - | 3.90E-04 | G(A) | -0.1(0.02) | 0.02(0.02) | 0.01(0.02) | 0.01(0.02) |
| rs1702294 | - | - | 8.66E-04 | C(T) | 0.12(0.01) | 0.01(0.02) | 0.01(0.02) | 0.01(0.02) |
| rs17194490 | - | - | 5.23E-04 | T(G) | 0.1(0.01) | -0.01(0.02) | 0.01(0.02) | -0.01(0.02) |
| rs2007044 | - | - | 8.73E-04 | G(A) | 0.09(0.01) | 0.02(0.01) | 0.02(0.02) | 0.03(0.02) |
| rs2053079 | - | - | 4.24E-04 | G(A) | 0.07(0.01) | 0.02(0.01) | 0.01(0.02) | 0.04(0.02) |
| rs2068012 | - | - | 3.64E-04 | C(T) | 0.07(0.01) | 0.03(0.01) | 0.03(0.02) | 0.03(0.02) |
| rs2514218 | - | - | 4.70E-04 | T(C) | -0.07(0.01) | -0.01(0.01) | -0.01(0.02) | -0.01(0.02) |
| rs2535627 | - | - | 5.33E-04 | C(T) | -0.07(0.01) | -0.01(0.01) | -0.01(0.02) | -0.01(0.02) |
| rs2693698 | - | - | 3.91E-04 | G(A) | 0.06(0.01) | 0.01(0.01) | 0.01(0.02) | 0.01(0.02) |
| rs2905426 | - | - | 4.07E-04 | T(G) | -0.06(0.01) | -0.04(0.01) | -0.04(0.02) | -0.04(0.02) |
| rs2909457 | - | - | 3.62E-04 | A(G) | -0.06(0.01) | -0.01(0.01) | -0.02(0.02) | 0.01(0.02) |
| rs2945232 | - | - | 3.82E-04 | C(T) | 0.06(0.01) | 0.01(0.01) | 0.02(0.02) | -0.01(0.02) |
| rs2973155 | - | - | 4.55E-04 | C(T) | 0.07(0.01) | 0.03(0.01) | 0.03(0.02) | 0.02(0.02) |
| rs301797 | - | - | 4.27E-04 | A(C) | 0.07(0.01) | 0.03(0.01) | 0.03(0.02) | 0.02(0.02) |
| rs34796896 | - | - | 5.20E-04 | A(G) | -0.09(0.01) | -0.03(0.02) | -0.01(0.02) | -0.04(0.02) |
| rs35225200 | - | - | 6.18E-04 | C(A) | 0.14(0.02) | 0.09(0.02) | 0.18(0.03) | 0.01(0.03) |
| rs36068923 | - | - | 5.07E-04 | G(A) | 0.08(0.01) | 0.01(0.02) | 0.01(0.02) | 0.02(0.02) |
| rs3735025 | - | - | 4.07E-04 | C(T) | -0.06(0.01) | -0.01(0.01) | 0.01(0.02) | 0.01(0.02) |
| rs3798869 | - | - | 4.54E-04 | A(G) | -0.06(0.01) | 0.01(0.01) | 0.01(0.02) | 0.01(0.02) |
| rs3849046 | - | - | 4.17E-04 | T(C) | 0.06(0.01) | -0.01(0.01) | -0.01(0.02) | -0.01(0.02) |
| rs4129585 | - | - | 6.52E-04 | C(A) | -0.08(0.01) | 0.01(0.01) | 0.01(0.02) | 0.01(0.02) |
| rs427230 | - | - | 3.68E-04 | T(C) | 0.12(0.02) | 0.04(0.02) | 0.04(0.03) | 0.04(0.03) |
| rs4391122 | - | - | 6.60E-04 | G(A) | 0.08(0.01) | -0.02(0.01) | -0.01(0.02) | -0.01(0.02) |
| rs4648845 | - | - | 4.22E-04 | T(C) | 0.07(0.01) | 0.01(0.01) | 0.03(0.02) | 0.02(0.02) |
| rs4702 | - | - | 5.95E-04 | A(G) | -0.08(0.01) | 0.03(0.01) | 0.04(0.02) | 0.01(0.02) |
| rs4766428 | - | - | 4.62E-04 | T(C) | 0.07(0.01) | 0.01(0.01) | -0.01(0.02) | 0.02(0.02) |
| rs56205728 | - | - | 3.61E-04 | A(G) | 0.07(0.01) | 0.03(0.01) | 0.03(0.02) | 0.03(0.02) |
| rs58120505 | - | - | 6.78E-04 | C(T) | -0.08(0.01) | -0.02(0.01) | -0.04(0.02) | -0.02(0.02) |
| rs59979824 | - | - | 3.98E-04 | A(C) | -0.07(0.01) | -0.01(0.01) | -0.01(0.02) | -0.02(0.02) |
| rs6065094 | - | - | 5.23E-04 | G(A) | 0.07(0.01) | 0.03(0.01) | 0.01(0.02) | 0.04(0.02) |
| rs6430095 | - | - | 3.92E-04 | A(G) | 0.08(0.01) | 0.04(0.02) | 0.03(0.02) | 0.04(0.02) |
| rs6704768 | - | - | 5.88E-04 | A(G) | -0.07(0.01) | 0.01(0.01) | 0.01(0.02) | -0.01(0.02) |
| rs6984242 | - | - | 4.40E-04 | A(G) | -0.06(0.01) | 0.02(0.01) | 0.03(0.02) | 0.01(0.02) |
| rs7193419 | - | - | 3.85E-04 | C(T) | -0.06(0.01) | 0.01(0.01) | 0.01(0.02) | 0.01(0.02) |
| rs72986630 | - | - | 3.61E-04 | T(C) | 0.15(0.03) | 0.01(0.03) | 0.02(0.04) | -0.01(0.04) |
| rs73036062 | - | - | 5.44E-04 | A(G) | -0.09(0.01) | 0.01(0.02) | 0.03(0.02) | -0.02(0.02) |
| rs73229090 | - | - | 3.84E-04 | A(C) | -0.1(0.02) | 0.01(0.02) | -0.01(0.03) | 0.01(0.03) |
| rs7405404 | - | - | 4.76E-04 | C(T) | -0.08(0.01) | -0.01(0.01) | -0.01(0.02) | -0.01(0.02) |
| rs7432375 | - | - | 5.21E-04 | A(G) | -0.07(0.01) | -0.01(0.01) | -0.01(0.02) | -0.01(0.02) |
| rs75968099 | - | - | 5.93E-04 | T(C) | 0.08(0.01) | 0.01(0.01) | -0.02(0.02) | 0.04(0.02) |
| rs77149735 | - | - | 4.18E-04 | A(G) | 0.28(0.05) | 0.03(0.05) | 0.04(0.07) | 0.04(0.07) |
| rs7819570 | - | - | 3.85E-04 | T(G) | 0.08(0.01) | 0.03(0.02) | 0.02(0.02) | 0.02(0.02) |
| rs7893279 | - | - | 5.31E-04 | G(T) | -0.11(0.02) | -0.01(0.02) | 0.02(0.03) | -0.01(0.03) |
| rs7951870 | - | - | 5.15E-04 | C(T) | 0.09(0.01) | -0.01(0.02) | 0.01(0.02) | -0.01(0.02) |
| rs8042374 | - | - | 6.05E-04 | G(A) | -0.09(0.01) | -0.01(0.01) | -0.01(0.02) | -0.01(0.02) |
| rs8044995 | - | - | 3.69E-04 | A(G) | 0.08(0.01) | -0.01(0.02) | -0.03(0.02) | 0.02(0.02) |
| rs8082590 | - | - | 4.07E-04 | A(G) | -0.07(0.01) | 0.01(0.01) | 0.01(0.02) | -0.01(0.02) |
| rs8139773 | - | - | 4.97E-04 | G(A) | 0.07(0.01) | 0.02(0.01) | 0.04(0.02) | 0.01(0.02) |
| rs832187 | - | - | 3.77E-04 | T(C) | -0.06(0.01) | -0.01(0.01) | 0.02(0.02) | -0.03(0.02) |
| rs9398171 | - | - | 3.73E-04 | T(C) | -0.06(0.01) | 0.01(0.01) | -0.01(0.02) | 0.01(0.02) |
| rs9636107 | - | - | 6.16E-04 | G(A) | 0.08(0.01) | 0.01(0.01) | 0.04(0.02) | -0.01(0.02) |

*Note.* All SNPs with P < 5×10^-8^ clumped at r^2^ < .001 with 10-Mb. SCZ: schizophrenia; IBD: inflammatory bowel disease; CD: Crohn's disease; UC: ulcerative colitis.

**Supplemental Table 2. Genetic instruments for IBD, CD and UC and associations with SCZ.**

| **Exposure** | **Proxy SNP** | **r2 for proxy** | **SNP** | **R^2^** | **A1 (A2)** | **β (SE) for exposures** | **β (SE) for SCZ** |
| --- | --- | --- | --- | --- | --- | --- | --- |
| IBD | - | - | rs10041497 | 6.72E-04 | C(T) | 0.08(0.01) | -0.01(0.01) |
| IBD | - | - | rs10114470 | 1.93E-03 | C(T) | 0.15(0.01) | 0.02(0.01) |
| IBD | - | - | rs10761659 | 2.63E-03 | G(A) | 0.16(0.01) | -0.03(0.01) |
| IBD | - | - | rs10800309 | 1.42E-03 | G(A) | -0.12(0.01) | 0.04(0.01) |
| IBD | - | - | rs10826797 | 8.83E-04 | T(G) | -0.1(0.01) | 0.01(0.01) |
| IBD | - | - | rs10953551 | 1.10E-03 | G(A) | -0.1(0.01) | 0.01(0.01) |
| IBD | - | - | rs11066188 | 7.53E-04 | A(G) | 0.09(0.01) | -0.01(0.01) |
| IBD | - | - | rs111456533 | 6.13E-04 | A(G) | -0.1(0.02) | 0.03(0.01) |
| IBD | - | - | rs11152949 | 9.78E-04 | G(A) | 0.1(0.01) | 0.02(0.01) |
| IBD | - | - | rs11195128 | 5.91E-04 | T(C) | 0.08(0.01) | -0.01(0.01) |
| IBD | - | - | rs11209013 | 6.48E-04 | G(A) | 0.08(0.01) | 0.01(0.01) |
| IBD | - | - | rs11221335 | 5.21E-04 | C(T) | 0.08(0.01) | 0.01(0.01) |
| IBD | - | - | rs11236797 | 2.36E-03 | A(C) | 0.15(0.01) | 0.02(0.01) |
| IBD | - | - | rs112936798 | 5.14E-04 | C(A) | -0.18(0.03) | 0.06(0.06) |
| IBD | - | - | rs1131095 | 2.59E-03 | C(T) | 0.16(0.01) | 0.02(0.01) |
| IBD | - | - | rs11548656 | 7.17E-04 | G(A) | -0.24(0.04) | 0.02(0.03) |
| IBD | - | - | rs11581607 | 8.28E-03 | A(G) | -0.66(0.03) | 0.01(0.02) |
| IBD | - | - | rs11669299 | 8.29E-04 | T(C) | -0.11(0.02) | 0.01(0.01) |
| IBD | - | - | rs11677002 | 9.10E-04 | C(T) | -0.09(0.01) | 0.03(0.01) |
| IBD | - | - | rs11734570 | 4.98E-04 | A(G) | 0.07(0.01) | 0.02(0.01) |
| IBD | - | - | rs11768365 | 5.05E-04 | G(A) | -0.08(0.02) | -0.01(0.01) |
| IBD | - | - | rs117981694 | 1.18E-03 | A(G) | 0.35(0.04) | 0.1(0.04) |
| IBD | - | - | rs1250573 | 8.40E-04 | A(G) | -0.1(0.01) | 0.01(0.01) |
| IBD | - | - | rs1268339 | 5.16E-04 | C(T) | 0.09(0.02) | 0.02(0.01) |
| IBD | - | - | rs12825700 | 1.81E-03 | A(G) | 0.13(0.01) | 0.01(0.01) |
| IBD | - | - | rs12936409 | 2.14E-03 | T(C) | 0.14(0.01) | -0.02(0.01) |
| IBD | - | - | rs1297264 | 2.24E-03 | G(A) | -0.15(0.01) | -0.01(0.01) |
| IBD | - | - | rs1317209 | 8.82E-04 | A(G) | 0.12(0.02) | 0.01(0.01) |
| IBD | - | - | rs1336900 | 7.32E-04 | A(G) | -0.08(0.01) | 0.02(0.01) |
| IBD | - | - | rs13422838 | 5.18E-04 | C(T) | -0.11(0.02) | -0.02(0.02) |
| IBD | - | - | rs140933577 | 6.18E-04 | C(T) | -0.19(0.03) | -0.03(0.02) |
| IBD | - | - | rs143210366 | 1.03E-03 | G(T) | 0.28(0.04) | 0.04(0.03) |
| IBD | - | - | rs1445004 | 2.94E-03 | T(C) | 0.17(0.01) | 0.01(0.01) |
| IBD | - | - | rs1456896 | 7.28E-04 | T(C) | 0.09(0.01) | 0.01(0.01) |
| IBD | - | - | rs149169037 | 5.10E-04 | A(G) | -0.13(0.02) | -0.01(0.02) |
| IBD | - | - | rs154873 | 6.32E-04 | A(G) | -0.08(0.01) | 0.01(0.01) |
| IBD | - | - | rs1558619 | 7.83E-04 | T(G) | -0.08(0.01) | 0.01(0.01) |
| IBD | - | - | rs16940202 | 7.45E-04 | C(T) | 0.11(0.02) | 0.01(0.01) |
| IBD | - | - | rs17656349 | 5.70E-04 | T(C) | 0.07(0.01) | -0.01(0.01) |
| IBD | - | - | rs194746 | 7.52E-04 | T(C) | 0.08(0.01) | 0.01(0.01) |
| IBD | - | - | rs212402 | 5.45E-04 | A(G) | -0.07(0.01) | -0.02(0.01) |
| IBD | - | - | rs2301127 | 6.44E-04 | A(G) | 0.08(0.01) | 0.01(0.01) |
| IBD | - | - | rs2384352 | 8.78E-04 | G(A) | 0.1(0.01) | 0.02(0.01) |
| IBD | - | - | rs2413583 | 1.71E-03 | T(C) | -0.17(0.02) | 0.01(0.01) |
| IBD | - | - | rs243505 | 6.59E-04 | G(A) | -0.08(0.01) | -0.01(0.01) |
| IBD | - | - | rs2593855 | 5.89E-04 | T(C) | -0.08(0.01) | 0.02(0.01) |
| IBD | - | - | rs2836881 | 2.11E-03 | T(G) | -0.16(0.01) | 0.01(0.01) |
| IBD | - | - | rs28374519 | 1.08E-03 | A(G) | -0.11(0.01) | -0.02(0.01) |
| IBD | - | - | rs2838517 | 1.75E-03 | C(T) | -0.13(0.01) | -0.01(0.01) |
| IBD | - | - | rs3024493 | 2.23E-03 | A(C) | 0.19(0.02) | -0.01(0.01) |
| IBD | - | - | rs341295 | 5.34E-04 | T(C) | 0.07(0.01) | 0.01(0.01) |
| IBD | - | - | rs35171809 | 1.30E-03 | G(A) | 0.11(0.01) | -0.01(0.01) |
| IBD | - | - | rs3792111 | 2.09E-03 | T(C) | 0.14(0.01) | 0.01(0.01) |
| IBD | - | - | rs3820330 | 6.77E-04 | A(C) | -0.09(0.01) | -0.01(0.01) |
| IBD | - | - | rs3829110 | 2.64E-03 | G(A) | 0.16(0.01) | 0.01(0.01) |
| IBD | - | - | rs3850378 | 9.18E-04 | C(T) | 0.15(0.02) | 0.01(0.02) |
| IBD | - | - | rs3897234 | 7.47E-04 | C(T) | 0.1(0.01) | 0.01(0.01) |
| IBD | - | - | rs4256018 | 5.41E-04 | G(T) | 0.08(0.01) | 0.03(0.01) |
| IBD | - | - | rs4276914 | 6.54E-04 | A(G) | 0.08(0.01) | 0.01(0.01) |
| IBD | - | - | rs4380956 | 8.50E-04 | A(G) | 0.09(0.01) | -0.01(0.01) |
| IBD | - | - | rs4676408 | 1.01E-03 | A(G) | 0.1(0.01) | 0.03(0.01) |
| IBD | - | - | rs4807569 | 1.18E-03 | C(A) | 0.13(0.02) | 0.04(0.01) |
| IBD | - | - | rs4957256 | 9.64E-04 | T(C) | -0.12(0.02) | 0.02(0.01) |
| IBD | - | - | rs503734 | 5.19E-04 | G(A) | -0.07(0.01) | -0.01(0.01) |
| IBD | - | - | rs55946629 | 8.67E-04 | A(C) | 0.13(0.02) | 0.01(0.02) |
| IBD | - | - | rs56062135 | 1.51E-03 | T(C) | 0.14(0.01) | 0.01(0.01) |
| IBD | - | - | rs56116661 | 6.27E-04 | T(C) | -0.1(0.02) | 0.01(0.01) |
| IBD | - | - | rs56235845 | 6.73E-04 | G(T) | 0.09(0.01) | -0.02(0.01) |
| IBD | - | - | rs5754100 | 1.09E-03 | C(T) | 0.13(0.02) | 0.01(0.01) |
| IBD | - | - | rs5763793 | 5.31E-04 | T(G) | 0.07(0.01) | 0.02(0.01) |
| IBD | - | - | rs6017342 | 1.22E-03 | C(A) | 0.12(0.01) | -0.02(0.01) |
| IBD | - | - | rs6062496 | 1.88E-03 | A(G) | 0.14(0.01) | -0.01(0.01) |
| IBD | - | - | rs6063502 | 5.00E-04 | G(A) | -0.07(0.01) | -0.01(0.01) |
| IBD | - | - | rs62126610 | 1.20E-03 | G(A) | 0.14(0.02) | 0.01(0.01) |
| IBD | - | - | rs62183956 | 6.49E-04 | T(C) | -0.08(0.01) | -0.01(0.01) |
| IBD | - | - | rs62324212 | 8.11E-04 | A(C) | 0.09(0.01) | 0.01(0.01) |
| IBD | - | - | rs62378712 | 4.98E-04 | C(T) | -0.08(0.01) | -0.01(0.01) |
| IBD | - | - | rs62408218 | 6.70E-04 | T(C) | -0.08(0.01) | -0.04(0.01) |
| IBD | - | - | rs62482552 | 5.28E-04 | A(G) | -0.07(0.01) | -0.03(0.01) |
| IBD | - | - | rs6579807 | 7.29E-04 | T(C) | 0.13(0.02) | -0.01(0.02) |
| IBD | - | - | rs6584282 | 2.50E-03 | G(A) | -0.15(0.01) | 0.01(0.01) |
| IBD | - | - | rs6740847 | 9.11E-04 | G(A) | -0.09(0.01) | -0.01(0.01) |
| IBD | - | - | rs6873866 | 8.59E-04 | C(T) | -0.09(0.01) | 0.03(0.01) |
| IBD | - | - | rs6933404 | 5.59E-04 | C(T) | 0.09(0.01) | 0.01(0.01) |
| IBD | - | - | rs714910 | 7.93E-04 | C(A) | -0.1(0.01) | -0.01(0.01) |
| IBD | - | - | rs7190426 | 5.28E-04 | C(A) | -0.09(0.02) | -0.01(0.01) |
| IBD | - | - | rs7256518 | 6.07E-04 | A(G) | -0.17(0.03) | -0.01(0.03) |
| IBD | - | - | rs72852162 | 5.21E-04 | C(A) | -0.11(0.02) | 0.01(0.02) |
| IBD | - | - | rs744166 | 1.29E-03 | G(A) | -0.11(0.01) | -0.01(0.01) |
| IBD | - | - | rs749910 | 3.36E-03 | A(G) | 0.2(0.01) | 0.01(0.01) |
| IBD | - | - | rs7532133 | 5.78E-04 | G(A) | 0.08(0.01) | 0.03(0.01) |
| IBD | - | - | rs755374 | 2.89E-03 | T(C) | 0.18(0.01) | 0.01(0.01) |
| IBD | - | - | rs7608697 | 2.04E-03 | C(A) | 0.14(0.01) | -0.02(0.01) |
| IBD | - | - | rs76286777 | 7.25E-04 | C(T) | 0.1(0.02) | -0.02(0.01) |
| IBD | - | - | rs7918084 | 5.38E-04 | T(C) | 0.07(0.01) | -0.01(0.01) |
| IBD | - | - | rs80262450 | 1.15E-03 | A(G) | 0.16(0.02) | -0.01(0.02) |
| IBD | - | - | rs938650 | 5.38E-04 | A(G) | -0.11(0.02) | 0.01(0.02) |
| IBD | - | - | rs9934775 | 7.02E-04 | T(C) | -0.11(0.02) | -0.01(0.01) |
| CD | - | - | rs10114470 | 2.25E-03 | C(T) | 0.17(1) | 0.02(0.01) |
| CD | - | - | rs1012636 | 1.05E-03 | T(G) | 0.13(1) | -0.02(0.01) |
| CD | - | - | rs10822050 | 3.15E-03 | C(T) | 0.18(1) | -0.04(0.01) |
| CD | - | - | rs10884966 | 1.09E-03 | A(G) | 0.11(1) | -0.01(0.01) |
| CD | - | - | rs11236797 | 2.96E-03 | A(C) | 0.18(1) | 0.02(0.01) |
| CD | - | - | rs112856973 | 1.09E-03 | C(T) | -0.16(1) | 0.03(0.02) |
| CD | - | - | rs114802258 | 8.48E-04 | T(C) | -0.22(1) | -0.01(0.02) |
| CD | - | - | rs1148246 | 1.56E-03 | T(C) | -0.13(1) | -0.02(0.01) |
| CD | - | - | rs11677002 | 1.18E-03 | C(T) | -0.11(1) | 0.03(0.01) |
| CD | - | - | rs11683692 | 7.90E-04 | C(T) | -0.21(1) | -0.02(0.02) |
| CD | - | - | rs12131079 | 9.70E-04 | T(C) | -0.11(1) | -0.03(0.01) |
| CD | - | - | rs1250573 | 1.79E-03 | A(G) | -0.15(1) | 0.01(0.01) |
| CD | - | - | rs12936409 | 1.97E-03 | T(C) | 0.14(1) | -0.02(0.01) |
| CD | - | - | rs1297264 | 2.92E-03 | G(A) | -0.18(1) | -0.01(0.01) |
| CD | - | - | rs13107325 | 1.24E-03 | T(C) | 0.2(1) | 0.15(0.02) |
| CD | - | - | rs1321859 | 9.23E-04 | T(C) | -0.1(1) | -0.04(0.01) |
| CD | - | - | rs1373904 | 1.38E-03 | G(A) | 0.14(1) | 0.04(0.01) |
| CD | - | - | rs144309607 | 1.55E-03 | T(C) | -0.37(1) | -0.02(0.03) |
| CD | - | - | rs1583792 | 7.54E-04 | T(C) | -0.09(1) | -0.05(0.01) |
| CD | - | - | rs181826 | 1.20E-03 | A(C) | 0.12(1) | -0.01(0.01) |
| CD | - | - | rs194746 | 9.10E-04 | T(C) | 0.1(1) | 0.01(0.01) |
| CD | - | - | rs2002695 | 1.16E-03 | G(A) | -0.13(1) | 0.01(0.01) |
| CD | - | - | rs2021511 | 8.77E-04 | T(C) | -0.11(1) | -0.01(0.01) |
| CD | - | - | rs2076756 | 1.20E-02 | G(A) | 0.39(1) | 0.01(0.01) |
| CD | - | - | rs212409 | 1.14E-03 | A(G) | -0.11(1) | -0.02(0.01) |
| CD | - | - | rs2143178 | 2.17E-03 | C(T) | -0.21(1) | 0.01(0.01) |
| CD | - | - | rs2188962 | 3.88E-03 | T(C) | 0.2(1) | -0.02(0.01) |
| CD | - | - | rs2284553 | 1.49E-03 | G(A) | 0.13(1) | 0.01(0.01) |
| CD | - | - | rs2838517 | 2.00E-03 | C(T) | -0.15(1) | -0.01(0.01) |
| CD | - | - | rs28999107 | 9.19E-04 | T(G) | 0.11(1) | 0.01(0.01) |
| CD | - | - | rs2948542 | 9.64E-04 | G(A) | 0.1(1) | -0.01(0.01) |
| CD | - | - | rs3122605 | 1.47E-03 | A(G) | -0.17(1) | 0.02(0.02) |
| CD | - | - | rs34004493 | 1.23E-03 | G(A) | 0.13(1) | -0.01(0.01) |
| CD | - | - | rs34635748 | 2.24E-03 | T(C) | 0.48(1) | 0.11(0.04) |
| CD | - | - | rs35171809 | 2.40E-03 | G(A) | 0.16(1) | -0.01(0.01) |
| CD | - | - | rs3761158 | 1.10E-03 | A(G) | -0.11(1) | -0.01(0.01) |
| CD | - | - | rs3816234 | 6.87E-03 | A(G) | 0.27(1) | 0.01(0.01) |
| CD | - | - | rs3850378 | 1.38E-03 | C(T) | 0.2(1) | 0.01(0.02) |
| CD | - | - | rs4077515 | 3.22E-03 | T(C) | 0.18(1) | 0.01(0.01) |
| CD | - | - | rs42861 | 1.37E-03 | G(A) | 0.12(1) | 0.02(0.01) |
| CD | - | - | rs4316387 | 1.16E-03 | C(T) | -0.13(1) | -0.02(0.01) |
| CD | - | - | rs4343432 | 1.19E-03 | G(A) | 0.11(1) | -0.01(0.01) |
| CD | - | - | rs4380956 | 1.59E-03 | A(G) | 0.13(1) | -0.01(0.01) |
| CD | - | - | rs4807570 | 2.18E-03 | A(G) | 0.18(1) | 0.04(0.01) |
| CD | - | - | rs492602 | 1.11E-03 | G(A) | 0.11(1) | 0.04(0.01) |
| CD | - | - | rs55946629 | 1.43E-03 | A(C) | 0.18(1) | 0.01(0.02) |
| CD | - | - | rs56116661 | 9.50E-04 | T(C) | -0.13(1) | 0.01(0.01) |
| CD | - | - | rs5754100 | 1.66E-03 | C(T) | 0.17(1) | 0.01(0.01) |
| CD | - | - | rs6062496 | 1.33E-03 | A(G) | 0.12(1) | -0.01(0.01) |
| CD | - | - | rs61839660 | 7.85E-04 | T(C) | 0.15(1) | 0.01(0.02) |
| CD | - | - | rs62126620 | 1.27E-03 | A(G) | 0.14(1) | 0.01(0.01) |
| CD | - | - | rs62324212 | 1.05E-03 | A(C) | 0.11(1) | 0.01(0.01) |
| CD | - | - | rs6451494 | 6.08E-03 | C(T) | 0.26(1) | 0.01(0.01) |
| CD | - | - | rs6579807 | 1.65E-03 | T(C) | 0.2(1) | -0.01(0.02) |
| CD | - | - | rs6584282 | 2.66E-03 | G(A) | -0.17(1) | 0.01(0.01) |
| CD | - | - | rs6679677 | 1.57E-03 | A(C) | -0.23(1) | 0.02(0.02) |
| CD | - | - | rs6704109 | 2.31E-03 | T(C) | 0.17(1) | 0.01(0.01) |
| CD | - | - | rs6740847 | 1.04E-03 | G(A) | -0.1(1) | -0.01(0.01) |
| CD | - | - | rs6808936 | 7.82E-04 | G(A) | 0.09(1) | 0.01(0.01) |
| CD | - | - | rs6873866 | 1.59E-03 | C(T) | -0.13(1) | 0.03(0.01) |
| CD | - | - | rs714910 | 1.77E-03 | C(A) | -0.15(1) | -0.01(0.01) |
| CD | - | - | rs72743461 | 2.01E-03 | A(C) | 0.17(1) | 0.01(0.01) |
| CD | - | - | rs72798422 | 5.11E-03 | C(T) | 0.55(1) | -0.01(0.03) |
| CD | - | - | rs73243877 | 7.48E-04 | G(A) | 0.12(1) | -0.01(0.01) |
| CD | - | - | rs73516754 | 1.76E-03 | C(A) | 0.14(1) | 0.02(0.01) |
| CD | - | - | rs744166 | 1.23E-03 | G(A) | -0.11(1) | -0.01(0.01) |
| CD | - | - | rs7517847 | 1.07E-02 | G(T) | -0.34(1) | 0.01(0.01) |
| CD | - | - | rs755374 | 3.17E-03 | T(C) | 0.2(1) | 0.01(0.01) |
| CD | - | - | rs7608697 | 1.41E-03 | C(A) | 0.12(1) | -0.02(0.01) |
| CD | - | - | rs77566919 | 8.60E-04 | A(G) | -0.11(1) | 0.02(0.01) |
| CD | - | - | rs80262450 | 2.14E-03 | A(G) | 0.23(1) | -0.01(0.02) |
| CD | - | - | rs938650 | 1.24E-03 | A(G) | -0.17(1) | 0.01(0.02) |
| CD | - | - | rs9482770 | 9.21E-04 | C(T) | 0.1(1) | -0.01(0.01) |
| CD | - | - | rs9656588 | 1.16E-03 | C(T) | 0.12(1) | 0.01(0.01) |
| CD | - | - | rs9836291 | 2.54E-03 | A(G) | 0.17(1) | 0.02(0.01) |
| UC | - | - | rs10272963 | 1.94E-03 | T(C) | -0.15(0.02) | -0.01(0.01) |
| UC | - | - | rs10408351 | 1.25E-03 | A(G) | 0.15(0.02) | -0.01(0.01) |
| UC | - | - | rs10761659 | 1.38E-03 | G(A) | 0.13(0.02) | -0.03(0.01) |
| UC | - | - | rs10817678 | 1.33E-03 | A(G) | 0.13(0.02) | 0.02(0.01) |
| UC | - | - | rs11209026 | 3.94E-03 | A(G) | -0.48(0.04) | 0.01(0.02) |
| UC | - | - | rs1131095 | 1.95E-03 | C(T) | 0.16(0.02) | 0.02(0.01) |
| UC | - | - | rs113986290 | 7.25E-04 | T(C) | -0.31(0.05) | 0.05(0.03) |
| UC | - | - | rs11651246 | 9.79E-04 | G(T) | 0.15(0.02) | 0.02(0.01) |
| UC | - | - | rs12825700 | 2.99E-03 | A(G) | 0.19(0.02) | 0.01(0.01) |
| UC | - | - | rs12936409 | 1.62E-03 | T(C) | 0.14(0.02) | -0.02(0.01) |
| UC | - | - | rs1317209 | 1.74E-03 | A(G) | 0.18(0.02) | 0.01(0.01) |
| UC | - | - | rs13200059 | 9.91E-04 | A(G) | 0.29(0.04) | 0.06(0.03) |
| UC | - | - | rs1359946 | 1.31E-03 | A(G) | 0.16(0.02) | 0.01(0.01) |
| UC | - | - | rs137845 | 8.90E-04 | G(A) | 0.1(0.02) | 0.04(0.01) |
| UC | - | - | rs16940186 | 8.74E-04 | C(T) | 0.14(0.02) | 0.01(0.01) |
| UC | - | - | rs1736161 | 1.26E-03 | A(G) | -0.12(0.02) | -0.01(0.01) |
| UC | - | - | rs17656349 | 6.96E-04 | T(C) | 0.09(0.02) | -0.01(0.01) |
| UC | - | - | rs17715902 | 7.48E-04 | A(G) | 0.1(0.02) | 0.01(0.01) |
| UC | - | - | rs2045241 | 8.60E-04 | A(G) | -0.11(0.02) | -0.01(0.01) |
| UC | - | - | rs2212434 | 1.35E-03 | T(C) | 0.13(0.02) | 0.02(0.01) |
| UC | - | - | rs2836881 | 3.08E-03 | T(G) | -0.22(0.02) | 0.01(0.01) |
| UC | rs28383314 | 0.90 | rs28383224 | 1.72E-03 | G(A) | -0.15(0.02) | 0.03(0.01) |
| UC | - | - | rs2838517 | 1.18E-03 | C(T) | -0.12(0.02) | -0.01(0.01) |
| UC | - | - | rs3024493 | 2.19E-03 | A(C) | 0.21(0.02) | -0.01(0.01) |
| UC | - | - | rs3812565 | 1.51E-03 | C(T) | 0.13(0.02) | -0.01(0.01) |
| UC | - | - | rs3820330 | 1.73E-03 | A(C) | -0.16(0.02) | -0.01(0.01) |
| UC | - | - | rs4676408 | 1.60E-03 | A(G) | 0.14(0.02) | 0.03(0.01) |
| UC | - | - | rs4728142 | 8.62E-04 | A(G) | 0.1(0.02) | -0.03(0.01) |
| UC | - | - | rs4993442 | 6.62E-04 | T(G) | -0.1(0.02) | -0.02(0.01) |
| UC | - | - | rs55905347 | 8.76E-04 | A(G) | 0.11(0.02) | 0.02(0.01) |
| UC | - | - | rs56062135 | 7.46E-04 | T(C) | 0.11(0.02) | 0.01(0.01) |
| UC | - | - | rs6017342 | 2.84E-03 | C(A) | 0.19(0.02) | -0.02(0.01) |
| UC | - | - | rs6062496 | 1.51E-03 | A(G) | 0.14(0.02) | -0.01(0.01) |
| UC | - | - | rs62180181 | 1.12E-03 | T(C) | 0.12(0.02) | 0.03(0.01) |
| UC | - | - | rs67111717 | 6.62E-04 | G(A) | 0.09(0.02) | -0.02(0.01) |
| UC | - | - | rs6889364 | 7.26E-04 | A(G) | 0.13(0.02) | -0.01(0.02) |
| UC | - | - | rs6933404 | 1.36E-03 | C(T) | 0.15(0.02) | 0.01(0.01) |
| UC | - | - | rs72704802 | 7.66E-04 | T(C) | -0.12(0.02) | -0.01(0.01) |
| UC | - | - | rs7523335 | 9.51E-04 | A(G) | -0.14(0.02) | 0.01(0.01) |
| UC | - | - | rs755374 | 2.18E-03 | T(C) | 0.17(0.02) | 0.01(0.01) |
| UC | - | - | rs7554511 | 1.44E-03 | A(C) | -0.14(0.02) | -0.05(0.01) |
| UC | - | - | rs7608697 | 2.14E-03 | C(A) | 0.16(0.02) | -0.02(0.01) |
| UC | - | - | rs78064630 | 7.09E-04 | A(G) | 0.18(0.03) | -0.01(0.02) |
| UC | - | - | rs79051659 | 8.03E-04 | A(G) | 0.16(0.03) | 0.02(0.02) |
| UC | - | - | rs7911117 | 6.85E-04 | G(T) | -0.13(0.02) | 0.04(0.02) |
| UC | - | - | rs7911680 | 2.00E-03 | C(A) | -0.15(0.02) | 0.01(0.01) |
| UC | - | - | rs798506 | 9.86E-04 | C(T) | -0.12(0.02) | 0.01(0.01) |
| UC | - | - | rs9271176 | 8.80E-03 | G(A) | -0.35(0.02) | -0.05(0.01) |
| UC | - | - | rs9611131 | 9.41E-04 | C(T) | -0.15(0.02) | 0.02(0.02) |
| UC | - | - | rs989960 | 1.25E-03 | T(C) | -0.12(0.02) | -0.01(0.01) |

*Note.* All SNPs with P < 5×10^-8^ clumped at r^2^ < .001 with 10-Mb. SCZ: schizophrenia; IBD: inflammatory bowel disease; CD: Crohn's disease; UC: ulcerative colitis.
